# COVID‐19 in cancer patients may be presented by atypical symptoms and higher mortality rate, a case‐controlled study from Iran

**DOI:** 10.1002/cnr2.1378

**Published:** 2021-03-20

**Authors:** Soodabeh Shahidsales, Seyed Amir Aledavood, Mona Joudi, Fatemeh Molaie, Habibollah Esmaily, Seyed Alireza Javadinia

**Affiliations:** ^1^ Cancer Research Centre Mashhad University of Medical Sciences Mashhad Iran; ^2^ Student Research Committee Mashhad University of Medical Sciences Mashhad Iran; ^3^ Department of Epidemiology and Biostatistics, School of Health Mashhad University of Medical Sciences Mashhad Iran; ^4^ Cellular and Molecular Research Center Sabzevar University of Medical Sciences Sabzevar Iran

**Keywords:** COVID‐19, malignancy, mortality

## Abstract

**Background:**

Coronavirus disease 2019 (COVID‐19) pandemic imposes serious problems to health systems around the world and its rapid expansion makes it difficult to serve patients with certain health conditions such as cancer patients which might be at high risk for mortality if they are infected by the severe acute respiratory syndrome coronavirus 2.

**Aim:**

To compare the outcomes of cancer patients admitted due to COVID‐19 and compare them with data of COVID‐19 infected patients without a history of cancer.

**Methods:**

In this case‐controlled study, 93 healthy people and 92 patients with malignancy admitted for COVID‐19 were enrolled. The clinical features and laboratory indicators were assessed at the presentation and both groups were followed‐up for treatment options and outcomes prospectively and compared at the level of *P* ≤ .05.

**Results:**

COVID‐19 related mortality rate in malignant patients was significantly higher than patients without malignancy (41.3% vs 17.2%, *P* = .0001). The risk of death increased significantly in patients with malignancy (OR = 8.4, *P* = .007) and mechanical ventilation (OR = 3.3, *P* = .034) independent of other variables. Fever (64.5% vs 43.5%, *P* = .004), chill (35.5% vs 14.1%, *P* = .001), malaise (49.5% and 30.4%, *P* = .008), dry cough (51.6% vs 26.1%, *P* = .0001), and vomiting (17.2% vs 5.4%, *P* = .012) were reported significantly lower in cancer patients.

**Conclusion:**

The results suggest that cancer patients who were infected by COVID‐19 may present with atypical symptoms are at higher risk of mortality independent of the demographic data, comorbidities, and treatments.

## 1 INTRODUCTION

Coronavirus disease 2019 (COVID‐19) is a severely contiguous infectious disease caused by severe acute respiratory syndrome coronavirus 2 (SARS‐CoV‐2) reported for the first time at the beginning of 2020 in Wuhan, China. However, less than 3 months later, the world health organization recognized its spread as a pandemic that influences all countries on different continents.^1^ In most clinical scenarios, COVID‐19 presented by mild to moderate self‐limiting flue like symptoms, however, in some certain populations, COVID‐19 infected patients experience the severe form of disease leading to a higher rate of mechanical ventilation, intensive care unit (ICU) admission, and mortality.^2^, ^3^ Patients with malignancy especially if they are on active anti‐tumor treatments are among this high‐risk population with a potential fivefold risk of severe events^4^, ^5^; however, the data in this context is conflicting and there is evidence clearly demonstrated no significant effect of recent anticancer therapies on mortality of COVID‐19.^6^, ^7^, ^8^ Since COVID‐19 is a new medical condition that most health care systems never faced before, data about different aspects of it, that is, high‐risk population, criteria for admission of patients, and treatment options are lacking. For example, in Iran, there is no specific report on the features of cancer patients with COVID‐19 and only two studies reported them as a part of the general single institutional studies.^9^, ^10^ Therefore, along with institutional‐based recommendations on the management of patients with cancer suffering from COVID‐19,^11^, ^12^ there is a need to assess the epidemiologic aspects of this medical situation. In this study, we aimed to assess the clinical features, laboratory values, treatment options, and outcomes of cancer patients admitted due to COVID‐19 and compare them with data of COVID‐19 infected patients without a history of cancer.

## 2 METHODS

This case‐control study was performed between February 20, 2020 and May 20, 2020 during the first peak of the COVID‐19 pandemic in COVID‐19 specific treatment centers of Mashhad, Iran. Mashhad is located in northeastern Iran and is the capital of Khorasan Razavi Province neighboring Afghanistan. With more than 6 million inhabitants, it is the second‐most‐populated city in Iran. Besides, this town is visited by millions of Iranian and international travelers monthly due to the presence of The Holy Shrine of Imam Reza.

The protocol of the study was approved by the Ethics Committee of Mashhad University of Medical Sciences (IR.MUMS.REC.1399.059) and a written informed consent form was obtained from the patients or the legal guardian. Medical documents of patients with malignancy admitted to the Imam Reza Educational Hospital, the Ghaem Educational Hospital, and the Shariati Educational Hospital are all affiliated to the Mashhad University of Medical Sciences because COVID‐19 were assessed, prospectively. COVID‐19 infected patients without previous history of malignancy from the same units were selected randomly and matched based on their gender and age. To perform a randomized selection of the non‐malignant patient, they were sorted based on the date of admission and then were enrolled based on the computer‐generated random number table.

The COVID‐19 was diagnosed based on the presence of signs and symptoms of severe respiratory illness plus the evidence of lung involvement on the high‐resolution CT (HRCT) of the chest with or without positive real‐time polymerase chain reaction (RT‐PCR) test for COVID‐19. The decision to include patients only based on their HRCT chest was primarily made because of the limited resources in Iran during the first peak of COVID‐19 and not performing the RT‐PCR for all patients and the potential false negative of this procedure. The demographic data and signs, symptoms, and laboratory tests result of patients at the presentation were recorded and patients were followed up until the death or discharge. The primary objectives of the study were treatment options, mechanical ventilation, ICU admission, and the final outcome. The time interval between the last oncologic treatment and the beginning of COVID‐19 was reported in patients on active oncologic treatments receiving (chemo+/−) radiotherapy or chemotherapy. The level of O_2_ saturation on pulse oximetry was grouped as normal, mild (O_2_sat = 90‐95%), moderate (O_2_sat = 90‐75%), and severe (O_2_sat < 75%) hypoxemia.

The sample size was estimated to be at least 33 COVID‐19 infected patients in each group based on the results of Erdal et al.^13^ who showed that the mortality rates of COVID‐19 infected patients with and without malignancy was 23.9% and 1.5%, respectively; using *n* = (*Zα*/2 + *Zβ*)2 * (*p*1(1 − *p*1) + *p*2(1 − *p*2))/(*p*1 − *p*2)2 with a confidence interval of 95% and poser of 90%.

Data were analyzed by SPSS‐21 using chi‐square and Fisher exact tests for categorical variables and independent *t*‐test for quantitative data (or Mann‐Whitney *U* test is a nonparametric test in case of the absence of normal distribution which was tested by Shapiro‐Wilk test) at the level of *P* ≤ .05. To assess the most parsimonious set of predictors that are most effective in predicting the death [odd ration (OR)], stepwise binary logistic regression was used.

## 3 RESULTS

Between 20 February 2020 and 20 May 2020, 3373 patients with the diagnosis of COVID‐19 were admitted at COVID‐19 specific treatment centers of Mashhad, Iran. In this research, 93 non‐malignant patients and 92 patients with malignancy admitted for COVID‐19 were enrolled. The rate of positive PCR for COVID‐19 was significantly higher in non‐malignant patients (64% vs 35.1%, *P*‐value = .0001). Comparing COVID‐19 infected malignant patients with COVID‐19 infected patients without malignancy, non‐malignant patients had higher rates of comorbidities (*P*‐value = .014) notably diabetes mellitus (*P*‐value = .006). COVID‐19 related mortality rate in malignant patients was significantly higher (41.3% vs 17.2%, *P*‐value = .0001), however, the cause of death was similar between the groups mostly due to acute respiratory distress syndrome (50% vs 60%), multiple organ dysfunction syndromes (41.7% vs 40%), and cardiac arrest (8.3% vs 0%) by *P*‐value = .483. The time interval to admission after the onset of COVID‐19 symptoms in malignant and non‐malignant patients was 7.16 ± 5.3 and 7.01 ± 7 days (*P*‐value = .089). Also, the duration of admission was the same (medians; 8 vs 6.5 days, *P*‐value = .155). Table 1 shows characteristics of COVID‐19 infected patients with and without malignancy in detail.

**TABLE 1:** Characteristics of COVID‐19 infected patients with and without malignancy

		Malignancy		*P*‐value
		No	Yes	
**Demographic data**				
Age [median, year]		57	62	.078
Gender	Male	56 (60.2%)	55 (59.8%)	.952
	Female	37 (39.8%)	37 (40.2%)	
Comorbidity		50 (53.8%)	33 (35.9%)	.014
Number of comorbidities	0	43 (46.2%)	59 (64.1%)	.064
	1	25 (26.9%)	20 (21.7%)	
	2	18 (19.4%)	7 (7.6%)	
	3	6 (6.5%)	6 (6.5%)	
	4	1 (1.1%)	0	
Diabetes mellitus		30 (32.3%)	14 (15.2%)	**.006**
Hypertension		28 (30.1%)	17 (18.5%)	.065
IHD/CHF		14 (15.1%)	14 (15.2%)	.975
COPD		4 (4.3%)	1 (1.1%)	.187
Asthma		2 (2.2%)	0	.251
CKD/ESRD		4 (4.3%)	2 (2.2%)	.346
Hepatitis B		0	2 (2.2%)	.246
Deep venous thrombosis		0	1 (1.1%)	.497
**Symptoms**				
Fatigue		66 (71%)	66 (71.7%)	.908
Fever		60 (64.5%)	40 (43.5%)	**.004**
Chill		33 (35.5%)	13 (14.1)	**.001**
Malaise		46 (49.5%)	28 (30.4%)	**.008**
Chest pain		10 (10.8%)	10 (10.9%)	.980
Headache		9 (9.7%)	7 (7.6%)	.617
Seizure		0	5 (5.4%)	.029
Lack of consciousness		3 (3.2%)	15 (16.3%)	**.003**
Dry cough		48 (51.6%)	24 (26.1%)	**.0001**
Productive cough		17 (18.3%)	18 (19.6%)	.823
Shortening of breath		82 (88.2%)	73 (79.3%)	.104
Hemoptysis		0	3 (3.3%)	.079
Sore throat		5 (5.4%)	2 (2.2%)	.227
Nausea		21 (22.6%)	13 (14.1%)	.138
Vomiting		16 (17.2%)	5 (5.4%)	**.012**
Diarrhea		6 (6.5%)	4 (4.3%)	.747
Constipation		1 (1.1%)	4 (4.3%)	.211
Gastrointestinal bleeding		0	4 (4.3%)	.059
Bowel obstruction		1 (1.1%)	1 (1.1%)	1
**Signs**				
Systolic blood pressure	[median, mmHg]	130	120	**.0001**
Diastolic blood pressure	[median, mmHg]	80	75	**.0001**
Oxygen saturation				
	Normal	11 (11.8%)	19 (20.9%)	.383
	O2sat = 90–95%	41 (41.1%)	33 (36.3%)	
	O2sat = 90–75%	37 (39.8%)	35 (38.5%)	
	O2sat < 75%	4 (4.3%)	4 (4.4%)	
Heart rate				
	Normal	58 (62.4%)	60 (65.2%)	.687
	Bradycardia	0	0	
	Tachycardia	35 (37.6%)	32 (34.8%)	
Respiratory rate				
	Normal	9 (9.7%)	5 (54%)	.275
	Bradypnea	0	0	
	Tachypnea	84 (90.3%)	87 (94.6%)	
Temperature				
	Normal	72 (77.4%)	81 (88%)	.102
	Hypothermia	1 (1.1%)	0	
	Fever	20 (21.5%)	11 (12%)	
**Laboratory data**				
Neutrophil count				
	Normal	64 (69.6%)	45 (48.9%)	**.002**
	Neutropenia	1 (1.1%)	11 (12%)	
	Neutrophilia	27 (29.3%)	36 (39.1%)	
Lymphocyte count				
	Normal	25 (26.9%)	32 (34.8%)	.245
	Lymphopenia	68 (73.1%)	60 (65.2%)	
Thrombocytopenia		7 (7.5%)	33 (35.9%)	**.0001**
Anemia		20 (21.5%)	34 (97%)	**.021**
Sodium level				
	Normal	64 (68.8%)	59 (64.1%)	.239
	Hyponatremia	28 (30.1%)	27 (29.3%)	
	Hypernatremia	1 (1.1%)	6 (6.6%)	
Potassium level				
	Normal	73 (78.5%)	68 (74.7)	.335
	Hypokalemia	18 (19.4%)	17 (18.7%)
	Hyperkalemia	2 (2.2%)	6 (6.6%)	
ESR [mean ± SD, mm/h]		54.5 ± 5.3	63.14 ± 4.8	.605
CRP [median, mg/L]		58.7	89.5	.109
Cr [median, mg/dL]		0.9	0.95	.802
SGOT [median, U/L]		30	33	.171
SGPT [median, U/L]		24.5	29	.404
LDH [median, U/L]		574.5	698.5	.075
Consolidation		54 (58.1%)	51 (57.3%)	.917
Ground glass opacity		87 (94.6%)	79 (88.8%)	.157
Location of lesions	Disseminated	60 (65.3%)	48 (53.9%)	.685
	Peripheral	21 (22.8)	12 (13.5%)	
	Bases	7 (7.6%)	19 (21.3%)	
	Apical	1 (1.1%)	7 (7.9%)	
	Peribronchovascular distribution	3 (3.3%)	3 (3.%)	
Bilaterality		88 (94.6%)	74 (82.2%)	**.009**
Pleural effusion		10 (10.8%)	36 (40.4%)	**.0001**
Lymphadenopathy		9 (9.7%)	20 (22.7%)	**.017**
Calcification		3 (3.2%)	3 (3.4%)	1
**COVID‐19 treatment data**				
O_2_ therapy		91 (97.8%)	82 (91.1%)	**.06**
Antibiotic therapy		92 (98.9%)	89 (98.9%)	1
Steroid therapy		12 (12.9%)	11 (12.4%)	1
Antiviral therapy		56 (60.2%)	51 (57.3%)	.690
Mechanical ventilation		10 (10.8%)	21 (23.6%)	**.021**
ICU admission		14 (15.1%)	22 (24.7%)	.102

*Note*: Bold numbers show the *p* values which are significant at the level of *p* < .05.

As shown in Table 2, most patients with malignancy suffered from hematologic and gastrointestinal cancers (33.3% and 24.7%) that 53.9% of them had metastatic disease. In patients on active treatment (except hormone therapy), the median time interval between the last oncologic treatment and COVID‐19 infection was 20 [95%CI 17‐29] days.

**TABLE 2:** The characteristics of patients with malignancy

	Frequency	Percent
**Type of cancer**		
Hematologic cancer	31	33.3
GI cancer	23	24.7
Lung cancer	9	9.7
Breast cancer	8	8.6
Urogenital cancers	6	6.5
Brain tumors	5	5.4
H&N cancer	5	5.4
GYN cancer	3	3.2
Melanoma	1	1.1
Sarcoma	1	1.1
Beast and endometrial cancer	1	1.1
**Stage**		
Metastatic	41	53.9
Nonmetastatic	33	43.9
Relapse	2	2.6
**Current status**		
Follow up	40	44.0
Chemotherapy	35	38.5
Radiotherapy	2	2.2
Targeted therapy	4	4.4
Hormone therapy	6	6.6
New case	4	4.4

Regression analysis showed that the risk of death increased significantly in patients with malignancy (OR = 8.4, *P* = .007) and mechanical ventilation (OR = 3.3, *P*‐value = .034) independent of other variables (Table 3).

**TABLE 3:** Stepwise binary logistic regression on variables predicting the death in patients with COVID‐19

	OR	95% CI		*P*‐value
Age	1.2	.977	1.079	.301
Gender (male)	1.9	.327	3.684	.881
Comorbidities (yes)	.8	.083	8.725	.893
Total number of comorbidities	1.6	.099	27.535	.727
DM (yes)	2	.130	31.423	.614
HTN (yes)	.3	.025	5.227	.456
IHD/CHF (yes)	.6	.022	17.620	.777
COPD (yes)	4	.062	259.718	.514
Malignancy (yes)	8.4	1.780	39.918	**.007**
Systolic blood pressure	1.1	.963	1.068	.599
Diastolic blood pressure	1.4	.928	1.086	.921
Moderate/severe hypoxemia (yes)	2.2	.071	12.101	.068
Tachycardia (yes)	.418	.112	1.552	.192
Tachypnea (yes)	.157	.004	6.577	.331
Fever (yes)	2.9	.309	28.004	.348
Neutropenia (yes)	.235	.013	4.277	.328
Neutrophilia (yes)	.685	.172	2.728	.591
Lymphopenia (yes)	2.8	.782	10.076	.113
Anemia (yes)	.201	.036	1.127	.068
Thrombocytopenia (yes)	.278	.065	1.196	.086
Bilateral lung involvement (yes)	.160	.017	1.477	.106
O_2_ therapy (yes)	.000	.000		.999
Antibiotic therapy (yes)	2.2	.042	119.259	.691
Steroid therapy (yes)	.223	.043	1.150	.073
Antiviral therapy (yes)	.698	.191	2.550	.587
Mechanical ventilation (yes)	3.3	1.52	10.35	**.001**
ICU admission (yes)	.269	.053	1.368	.114

*Note*: Bold numbers show the *p* values which are significant at the level of *p* < .05.

Subsequently, the stepwise binary logistic regression was performed in COVID‐19 infected patients with cancer to define the predisposing factors of death. However, no factor can predict the outcome including the type of malignancy, stage of the disease, and recent oncologic treatment (chemotherapy or radiotherapy, data not presented).

## 4 DISCUSSION

This study aimed to assess the clinical features, laboratory values, treatment options, and outcomes of cancer patients admitted due to COVID‐19 and compare them with data of COVID‐19 infected patients without a history of cancer. Our results showed that mortality was significantly higher in cancer patients infected by COVID‐19, although the comorbidities especially diabetes mellitus were more prevalent in non‐malignant patients. Moreover, the probability of positive PCR for CODIV‐19 was significantly higher in non‐malignant patients. Regression analysis showed that the risk of death in COVID‐19 infected patients with malignancy was about nine times more than other patients. Also, the patients who needed mechanical ventilation had a significantly higher mortality rate. An overview of recent data on COVID‐19 and its impact on patients with malignancies has been provided in Table 4.

**TABLE 4:** An overview of recent data on COVID‐19 and its impact on patients with malignancies

Authors	Year	Country	Population	ICU admission	Mortality rate
Guan et al.^14^	2019	China	Non‐cancer (1089) Cancer (10)	4.8% 30%	1.4% 0
Huang et al.^15^	2020	China	Non‐cancer (40) Cancer (1)	31.7% 0	15% 0
Yang et al.^16^	2020	China	Non‐cancer (50) Cancer (2)	100% 100%	62% 50%
Wang et al.^17^	2020	China	Non‐cancer (128) Cancer (10)	25% 40%	—
Lei et al.^18^	2020	China	Non‐cancer (25) Cancer (9)	40% 55.5%	12% 44.4%
Lee et al.^19^	2020	UK	Cancer (1044)	—	28.2%
Erdal et al.^13^	2021	Turkey	Non‐cancer (4412) Cancer (77)	—	1.51% 23.9%
Present study	2020	Iran	Non‐cancer (93) Cancer (92)	15.1% 24.7%	17.2% 41.3%

As stated earlier, there are limited data on the potential interaction of COVID‐19 and malignancy and its treatments, and this specific category of COVID‐19 infected patients accounts for a small portion of patients in the recent studies.^14^, ^15^, ^20^ However, studies from China, Europe, and the United States proposed the predisposing role of malignancy on the increased mortality rate of COVID‐19 with higher rates for patients with active cancer receiving the anti‐cancer treatments.^4^, ^7^, ^19^, ^21^ Although, there is not a general agreement in this context with lower mortality rates in cancer patients with COVID‐19.^6^, ^17^

Our study showed that cancer patients might present less with typical symptoms of COVID‐19 and COVID‐19 infected patients with malignancy reported a lower frequency of fever and dry cough comparing previous studies.^5^, ^18^ However, studies are confirming our results regarding obscuring the main presentation of COVID‐19 in cancer patients.^22^ Also, the rate of neutropenia, anemia, and thrombocytopenia which mainly had been the side‐effects of chemotherapy was higher in our study as expected. Besides, pleural effusion and lymphadenopathy were reported significantly higher in patients with malignancy that we believed is related to the underlying malignant condition.

Our study has some limitations. Due to limited resources during the first peak of pandemic and special economic conditions of Iran, it was not possible to do PCR tests for all patients and therefore, we had to enroll patients only based on pulmonary symptoms and radiography. Also, our analyses were based on patients with symptomatic cancer admitted to COVID‐19 specific treatment centers of Mashhad, and patients who managed in an outpatient setting or were asymptomatic were not included. Therefore, the cohort might not be entirely representative of all patients with cancer. Patients on an end‐of‐life care pathway would be unlikely to be included in the current study.

## 5 CONCLUSION

The results suggest that cancer patients who were infected by COVID‐19 are at higher risk of mortality independent of the demographic data, comorbidities, and treatments. Also, the false‐negative rate of PCR may be higher in COVID‐19 infected patients with malignancy and they might present with atypical presentation leading to delay diagnosis, collectively.

## AUTHOR CONTRIBUTIONS

**Soodabeh Shahidsales:** Conceptualization; project administration; supervision; validation. **Seyed Amir Aledavood:** Investigation; methodology; writing‐review & editing. **Mona Joudi:** Investigation; methodology; project administration; visualization; writing‐review & editing. **Fatemeh Molaie:** Conceptualization; data curation; formal analysis; investigation; methodology. **Habibollah Esmaeili:** Formal analysis; methodology; software; visualization; writing‐original draft. **Seyed Alireza Javadinia:** Conceptualization; formal analysis; investigation; methodology; writing‐original draft; writing‐review & editing.

## CONFLICT OF INTEREST

The authors declare that there is no conflict of interest to be reported.

## ETHICAL STATEMENT

The protocol of the study was approved by the Ethics Committee of Mashhad University of Medical Sciences (IR.MUMS.REC.1399.059) and a written informed consent form was obtained from the patients or the legal guardian.

## Data Availability

All data generated and analyzed during this study can be accessible through direct communication with corresponding author and agreement of all research team members.

## References

[1] World Health Organization . Coronavirus Disease (COVID‐19): Weekly Epidemiological Update. 2020. https://www.who.int/publications/m/item/covid-19-weekly-epidemiological-update.

[2] Onder G , Rezza G , Brusaferro S . Case‐fatality rate and characteristics of patients dying in relation to COVID‐19 in Italy. JAMA. 2020;323(18):1775‐1776. 10.1001/jama.2020.4683. 32203977

[3] Zhang J , Wang X , Jia X , et al. Risk factors for disease severity, unimprovement, and mortality in COVID‐19 patients in Wuhan, China. Clin Microbiol Infect. 2020;26(6):767‐772. 10.1016/j.cmi.2020.04.012. 32304745 PMC7159868

[4] Liang W , Guan W , Chen R , et al. Cancer patients in SARS‐CoV‐2 infection: a nationwide analysis in China. Lancet Oncol. 2020;21(3):335‐337. 32066541 10.1016/S1470-2045(20)30096-6 PMC7159000

[5] Zhang L , Zhu F , Xie L , et al. Clinical characteristics of COVID‐19‐infected cancer patients: a retrospective case study in three hospitals within Wuhan, China. Ann Oncol. 2020;31(7):894‐901. 10.1016/j.annonc.2020.03.296. 32224151 PMC7270947

[6] Kuderer NM , Choueiri TK , Shah DP , et al. Clinical impact of COVID‐19 on patients with cancer (CCC19): a cohort study. Lancet. 2020;395(10241):1907‐1918. 10.1016/s0140-6736(20)31187-9. 32473681 PMC7255743

[7] Lee LY , Cazier JB , Angelis V , et al. COVID‐19 mortality in patients with cancer on chemotherapy or other anticancer treatments: a prospective cohort study. Lancet. 2020;395(10241):1919‐1926. 10.1016/S0140-6736(20)31173-9. Epub 2020 May 28. Erratum in: Lancet. 2020 Aug 22;396(10250):534. PMID: 32473682; PMCID: PMC7255715. 32473682 PMC7255715

[8] Vuagnat P , Frelaut M , Ramtohul T , et al. COVID‐19 in breast cancer patients: a cohort at the Institut Curie Hospitals in the Paris area. Breast Cancer Res. 2020;22:1‐10. 10.1186/s13058-020-01293-8 PMC7254663 32460829

[9] Alamdari NM , Afaghi S , Rahimi FS , et al. Mortality risk factors among hospitalized COVID‐19 patients in a major referral center in Iran. Tohoku J Exp Med. 2020;252(1):73‐84. 32908083 10.1620/tjem.252.73

[10] Nikpouraghdam M , Jalali Farahani A , Alishiri G , et al. Epidemiological characteristics of coronavirus disease 2019 (COVID‐19) patients in IRAN: a single center study. J Clin Virol. 2020;127:104378. 10.1016/j.jcv.2020.104378. 32353762 PMC7172806

[11] Rakhsha A , Azghandi S , Taghizadeh‐Hesary F . Decision on chemotherapy amidst COVID‐19 pandemic: a review and a practical approach from Iran. Infect Chemother. 2020;52(4):496‐502. 10.3947/ic.2020.52.4.496. 33263246 PMC7779976

[12] Siavashpour Z , Taghizadeh‐Hesary F , Rakhsha A . Recommendations on management of locally advanced rectal cancer during the COVID‐19 pandemic: an Iranian consensus. J Gastrointest Cancer. 2020;51(3):800‐804. 10.1007/s12029-020-00454-4. 32656628 PMC7355082

[13] Erdal GS , Polat O , Erdem GU , et al. The mortality rate of COVID‐19 was high in cancer patients: a retrospective single‐center study. Int J Clin Oncol. 2021;1‐9. 10.1007/s10147-021-01863-6. Epub ahead of print. PMID: 33486624; PMCID: PMC7826293. 33486624 PMC7826293

[14] Guan WJ , Ni ZY , Hu Y , et al. Clinical characteristics of coronavirus disease 2019 in China. N Engl J Med. 2020;382(18):1708‐1720. 10.1056/NEJMoa2002032. 32109013 PMC7092819

[15] Huang C , Wang Y , Li X , et al. Clinical features of patients infected with 2019 novel coronavirus in Wuhan, China. Lancet. 2020;395(10223):497‐506. 10.1016/S0140-6736(20)30183-5. 31986264 PMC7159299

[16] Yang X , Yu Y , Xu J , et al. Clinical course and outcomes of critically ill patients with SARS‐CoV‐2 pneumonia in Wuhan, China: a single‐centered, retrospective, observational study. Lancet Respir Med. 2020;8(5):475‐481. 10.1016/s2213-2600(20)30079-5. 32105632 PMC7102538

[17] Wang D , Hu B , Hu C , et al. Clinical characteristics of 138 hospitalized patients with 2019 novel coronavirus‐infected pneumonia in Wuhan, China. JAMA. 2020;323(11):1061‐1069. 10.1001/jama.2020.1585. 32031570 PMC7042881

[18] Lei S , Jiang F , Su W , et al. Clinical characteristics and outcomes of patients undergoing surgeries during the incubation period of COVID‐19 infection. EClinicalMedicine. 2020;21:100331. 10.1016/j.eclinm.2020.100331. 32292899 PMC7128617

[19] Lee LYW , Cazier J‐B , Starkey T , et al. COVID‐19 prevalence and mortality in patients with cancer and the effect of primary tumour subtype and patient demographics: a prospective cohort study. Lancet Oncol. 2020;21(10):1309‐1316. 10.1016/S1470-2045(20)30442-3. 32853557 PMC7444972

[20] Chen N , Zhou M , Dong X , et al. Epidemiological and clinical characteristics of 99 cases of 2019 novel coronavirus pneumonia in Wuhan, China: a descriptive study. Lancet. 2020;395(10223):507‐513. 10.1016/S0140-6736(20)30211-7. 32007143 PMC7135076

[21] Passamonti F , Cattaneo C , Arcaini L , et al. Clinical characteristics and risk factors associated with COVID‐19 severity in patients with haematological malignancies in Italy: a retrospective, multicentre, cohort study. Lancet Haematol. 2020;7(10):e737‐e745. 10.1016/S2352-3026(20)30251-9. 32798473 PMC7426107

[22] Yang F , Shi S , Zhu J , Shi J , Dai K , Chen X . Clinical characteristics and outcomes of cancer patients with COVID‐19. J Med Virol. 2020;92(10):2067‐2073. 10.1002/jmv.25972. 32369209

